# Gap in knowledge of health benefits and risks of combined oral contraceptives among Lebanese women

**DOI:** 10.1186/s12889-023-17439-0

**Published:** 2024-01-02

**Authors:** Maroun J. Ibrahim, Lynn E. Khalife, Yara D. Ghanem, Ghady S. Baz, Michele M. Cherfane

**Affiliations:** 1 Gilbert and Rose-Marie Chagoury School of Medicine, Lebanese American University, Byblos, Lebanon, Level 4, Office 4702, Byblos, P.O. Box 36, Lebanon; 2 INSPECT-LB (Institut National de Santé Publique, d’Épidémiologie Clinique et de Toxicologie-Liban), Beirut, Lebanon

**Keywords:** Oral contraceptive pills, Women’s health, Lebanon, Knowledge, Benefits, Risks, Information

## Abstract

**Background:**

Oral Contraceptive Pills (OCPs) are among the most commonly used forms of contraception, but they are associated with several health benefits and risks. This study aims to determine the gap in knowledge of the underlying health benefits and risks of OCPs among Lebanese women and to identify the factors that might influence their beliefs.

**Methods:**

A questionnaire was completed by 817 Lebanese women aged 18–64 years old and assessed sociodemographic details, medical information, contraceptive practices, knowledge of underlying health benefits and risks, and information needs related to OCPs.

**Results:**

Among the total participants, 41.5% of women reported using OCPs at some point in their lives yet 46.6% denied receiving information about their benefits and 48% denied receiving information about their risks. The mean total OCP knowledge score was 5.70 out of 25, the mean OCP risk knowledge score was 4.09 out of 15, and the mean OCP benefit knowledge score was 0.77 out of 6. Sociodemographic factors associated with greater total knowledge, risk knowledge and benefit knowledge included OCP usage, being a student, confidence in one’s knowledge and satisfaction with one’s information. Both the total and risk knowledge scores were found to be higher in women who found that receiving information related to OCPs was important. Finally, participants who lived in central governates had greater total knowledge scores, whereas those with higher levels of education and a family history of endometrial cancer demonstrated better benefit knowledge.

**Conclusions:**

This study highlighted the poor knowledge of health benefits and risks associated with OCP use among Lebanese women and the associated sociodemographic factors that might influence their beliefs.

## Background

Oral Contraceptive Pills (OCPs) are not only used for family planning and pregnancy prevention [1,2] but also for their non-contraceptive benefits like in the setting of hormonal imbalances and menstrual and gynecological disorders [3–5]. In addition, OCPs have been shown to have protective roles against endometrial cancer and ovarian cancer [6–9] that a majority of women are not aware of, as shown in a Canadian study [10]. On the other hand, they have significant health risks including venous thromboembolism, hypertension, stroke, cervical cancer, and possibly breast cancer [4,11,12]. Interestingly, a study in Saudi Arabia showed that 61.6% of uneducated women and 51.4% of educated women did not consider thromboembolism as a side effect of OCPs [13]. Furthermore, previous studies showed that many women have refrained or discontinued OCPs for reasons like concerns about side effects, [14–16] the desire to conceive, [2] inconvenience and forgetfulness, [17–19] religious constraints, [20] cultural restrictions, [21] as well as fear of fertility changes [19]. In addition, some beliefs that women have about OCPs including fear of subfertility after discontinuation or weight gain have been reported as misconceptions due to their lack of scientific evidence [22,23]. This was noted in a study in Turkey that reported that 13.4% of women believed such misconceptions while another 41.9% were not sure of the risk of infertility due to OCPs [22].

These beliefs can be explained by an inadequate knowledge [4,11,12] which can promote the haphazard use of OCPs and amplify the underlying health risks and side effects [18,24]. Studies addressing knowledge, attitude, and practice towards OCPs have been conducted in many countries and have highlighted the influence of socio-demographic, cultural, and educational differences on those variables and most have found a positive correlation between OCP knowledge and higher educational levels, being married, longer duration of use and older age [2,12,18,25,26]. Research regarding knowledge about the benefits and harms of OCPs is minimal in the Arab region [24] and when it comes to Lebanon, previous studies have evaluated the extent of knowledge and the practice of contraception exclusively among Lebanese university students [5,27]. However, limited data is found on Lebanese women’s awareness on the potential benefits and harms of OCPs. In addition, no prior research in Lebanon has evaluated the differences between the perceived knowledge and actual knowledge regarding OCPs and the sociodemographic factors that might influence them.

Given that poor knowledge can be associated with negative outcomes on women and reproductive health, we conducted this study to assess the knowledge of Lebanese women regarding the health benefits and risks associated with OCPs and to determine the factors that may influence their beliefs.

## Materials and methods

### Study design

We conducted a cross-sectional study over a period of 3 months, from January 2023 to March 2023, to assess the knowledge of health benefits and risks of OCPs among Lebanese women.

### Participants and sample size

Women aged 18–65, with Lebanese citizenship and residing in Lebanon are eligible to participate in this study, with no a priori exclusion criteria. The sample size was calculated using Epi Info™ (Center for Disease Control, Atlanta, GA, USA. Available from: http://wwwn.cdc.gov/epiinfo). Based on the 2022 population statistics (Available from: http://cas.gov.lb), the estimated population in Lebanon is 6,825,445 with 80% being Lebanese. From a total of 5,460,356 Lebanese, 51.1% are females of which 56.4% are between 19 and 64 years of age. This yields a target population of approximately 1,618,340 Lebanese women. With a 95% confidence interval (CI) and a ± 3.5% margin of error, a minimum sample size of 784 participants is needed.

### Procedure

The Institutional Review Board at the Lebanese American University gave approval for the study (LAU.SOM.MC1.19/Dec/2022), and informed consent was obtained from all participants. Participation was voluntary, anonymous, and confidential. Detailed information and explanation of the scope and objectives of the study were included in the invitation to participate. Collected data was encrypted and downloaded on password protected computers and was available as de-identified electronic data in Microsoft Excel spreadsheets and SPSS files.

### Materials

Data was collected through an anonymous online questionnaire via Google Forms constructed based on “Women’s Knowledge Beliefs and Information Needs in Relation to the Risks and Benefits Associated With Use of The Oral Contraceptive Pill” [6] after receiving approval from one of the authors.

The questionnaire was available in both English and Arabic and was electronically distributed via social media platforms such as emails, WhatsApp groups and Instagram.

Before administration, the questionnaire was pilot-tested with a sample of 20 Lebanese women to identify any ambiguities or issues in the questions.

Then, with the help of an expert in reproductive health, the questionnaire was reviewed to ensure it covers the relevant content adequately. As such, the questionnaire was tailored to address the cultural nuances of our study population.

Initially, sociodemographic, medical characteristics and information about the practice of OCPs were gathered. Participants were then asked whether they have received information related to OCPs and discussed using them with a health professional. The perceived knowledge of the health benefits and risks of OCPs was assessed with a 5-point scale and the actual knowledge was determined by asking participants to indicate whether they thought OCP use decreased, increased, or did not affect the risk of 25 different medical conditions. Finally, feedback on information needs related to OCPs was determined by assessing the perceived importance on receiving information with a 5-point scale, their main source of information, the satisfaction with their information with a 5-point scale, and their preferred methods for receiving information.

Knowledge was defined as the ability to correctly identify the health benefits and risks of OCPs. A total knowledge score, a benefit knowledge score and a risk knowledge score were computed and accounted for 1 point for the correct answer and no point for an incorrect answer. The total knowledge score ranged from 0 to 25, the benefit knowledge score ranged from 0 to 6 and the risk knowledge score ranged from 0 to15 with higher values indicating better OCP knowledge.

### Statistical analysis

The collected data was available as de-identified electronic data in Microsoft Excel spreadsheets and SPSS files.

Descriptive statistics were performed using frequencies and percentages for categorical variables and means and standard deviations for continuous variables.

The mean knowledge scores were determined, and bivariate analysis was performed to determine differences in the knowledge and attitudes of participants towards OCPs according to sociodemographic characteristics. The student t-test was used to compare two means of continuous variables between dichotomous groups, ANOVA to compare between three or more means and the chi-square test to compare percentages. All variables that showed a *p*-value of < 0.25 in the bivariate analysis were included in the multivariable analyses to avoid potential confounders. Separate multivariable analyses to determine key factors associated with better knowledge among respondents were done using linear regression analysis and three models were analyzed for the total knowledge, health risks knowledge and health benefits knowledge scores, respectively. Data was presented along with 95% confidence intervals and a *p* value of < 0.05 was considered statistically significant.

## Results

### Sociodemographic characteristics

A total of 852 Lebanese women participated in this study, of whom 35 were excluded due to incompatibility with the inclusion criteria thus yielding a total of 817 participants between the ages of 18 and 64 who were willing to participate after filling an informed consent.

The sociodemographic characteristics are summarized in Table 1.

**Table 1:** Sociodemographic characteristics and medical information of sample (N = 817)

*Descriptive parameter*	*N (%)*
**Age in years**	
* 18–29*	*387 (47.4%)*
* 30–49*	*322 (39.5%)*
*50–64*	*107 (13.1%)*
**Marital status**	
*Single*	*373 (45.7%)*
*Married*	*410 (50.2%)*
*Separated-Widowed-Divorced*	*34 (4.2%)*
**Occupation**	
*Student*	*254 (31.1%)*
*Employed-Self employed*	*372 (45.5%)*
*Housewife - Retired*	*191 (23.4%)*
**Governate**	
*Beirut*	*105 (12.9%)*
*Bekaa and Baalbeck El Hermel*	*34 (4.2%)*
*Mount Lebanon*	*317 (38.8%)*
*North Lebanon and Akkar*	*245 (30%)*
*South Lebanon*	*116 (14.2%)*
**Highest attained education**	
*High School and less*	*241 (29.5%)*
*Bachelor’s Degree*	*324 (39.7%)*
*Post- graduate Degree*	*252 (30.8%)*
**Monthly personal income**	
*Less than 200$*	*246 (30.1%)*
*200$-400$*	*116 (14.2%)*
*400–1000$*	*118 (14.4%)*
*More than 1000$*	*91 (11.1%)*
*Prefers not to say*	*246 (30.1%)*
**Parental status**	
*Has children*	*410 (50.2%)*
*Does not have children*	*407 (49.8%)*
**Self-reported family history of cancer**	
*Breast cancer*	*216 (26.4%)*
*Endometrial cancer*	*26 (3.2%)*
*Ovarian cancer*	*53 (6.5%)*
**Self-reported family history of heart disease**	*454 (55.6%)*

### Practice of OCPs

Among the total participants, 339 (41.5%) of women reported OCP use at some point in their lives. The mean age of onset of OCP use was 24 years (SD = 6.7) with approximately one third of participants using them for over 6 months (35.7%). The main reasons for OCP use (N = 339) were for menstrual cycle regulation (48.4%) followed by pregnancy prevention (44%). Most participants who discontinued (33%) or stopped (30.8%) OCPs were concerned about side effects. The mains reasons for OCP use, discontinued use, and nonuse are summarized in Table 2.

**Table 2:** Reasons for oral contraceptive pill use, discontinuation of use and non-use

*Descriptive parameter*	*N (%)*
**Reasons for use of OCPs**	***N = 339***
*Pregnancy prevention*	*149 (44%)*
*Menstrual cycle regulation*	*164 (48.4%)*
*Non-contraceptive reasons (PCOS, endometriosis, menstrual pain…)*	*117 (34.5%)*
*Others*	*13 (3.8%)*
*Only form of contraception aware of*	*6 (1.8%)*
**Reasons for discontinuation of OCPs**	***N = 339***
*Attempting to get pregnant*	*39 (11.5%)*
*Belief that it is healthy to stop for a while*	*43 (12.7%)*
*Concerns about side effects*	*112 (33%)*
*Difficulty with compliance*	*13 (3.8%)*
*Limited access*	*9 (2.7%)*
*Friend or family recommendation*	*4 (1.2%)*
*Resorted to other forms of contraception*	*42 (12.4%)*
*Not Applicable*	*66 (19.5%)*
*Doctor Prescription*	*36 (10.6%)*
*Regulated Menstruation*	*13 (3.8%)*
*Side Effects*	*20 (5.9%)*
*Others*	*7 (2.1%)*
**Reasons for never having used OCPs**	***N = 478***
*Religious or cultural reasons*	*30 (6.3%)*
*Concerns about side effects*	*147 (30.8%)*
*Belief that it is unnatural or unhealthy*	*61 (12.8%)*
*Infertile*	*1 (0.2%)*
*Concerns about compliance*	*15 (3.1%)*
*Limited access*	*10 (2.1%)*
*Medical condition*	*13 (2.7%)*
*Sexual abstinence*	*46 (9.6%)*
*Partner opposition*	*5 (1%)*
*Others forms of contraception*	*9 (1.9%)*
*Not applicable*	*234 (39%)*

Total is more than 100% because some women reported several reasons

### Information related to oral contraceptive pills

Among responders (N = 817), 46.6% denied receiving information about the health benefits of OCPs and 48% denied receiving information about the health risks of OCPs. Most participants (53%) reported never discussing OCP use with healthcare professionals although the main source of information was from healthcare professionals (42.1%) and the majority preferred receiving information through medical consultations (66%).

### Self-assessment of knowledge of health benefits and health risks of OCPs

**Fig. 1:**
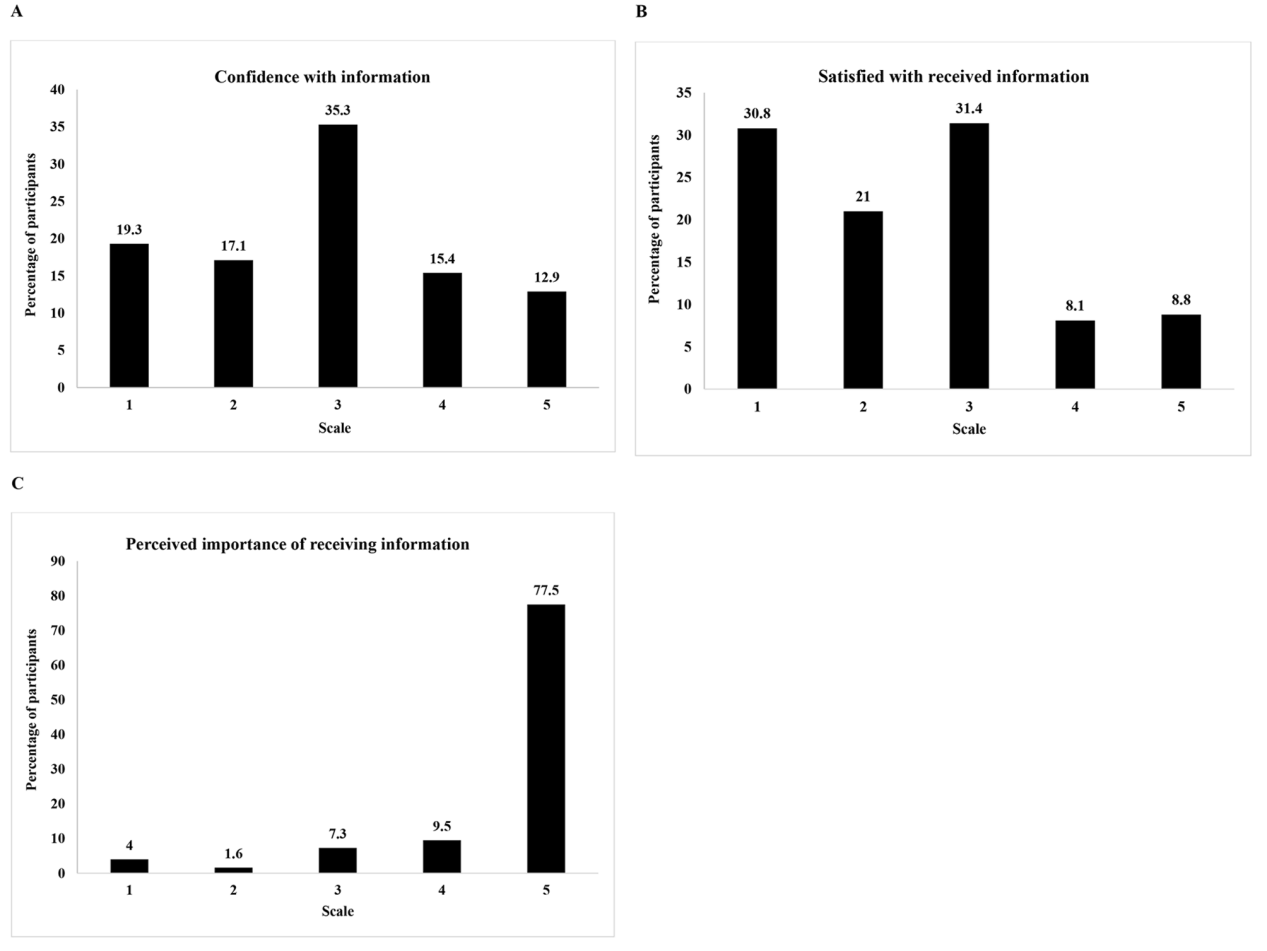
Confidence (**A**), perceived importance (**B**), and satisfaction with information (**C**) regarding OCPs on a scale from 1 to 5 (N = 817)

The mean knowledge score was 5.70 (SD = 4.88) out of 25, the mean risk knowledge score was 4.09 (SD = 3.88) out of 15, and the mean benefit knowledge score was 0.77 (SD = 1.17) out of 6. Furthermore, 35.3% of participants felt fairly confident with their knowledge, more than 60% indicated they were not satisfied with their knowledge but 77.5% of women found that receiving information related to OCPs was extremely important (Fig. 1).

**Fig. 2:**
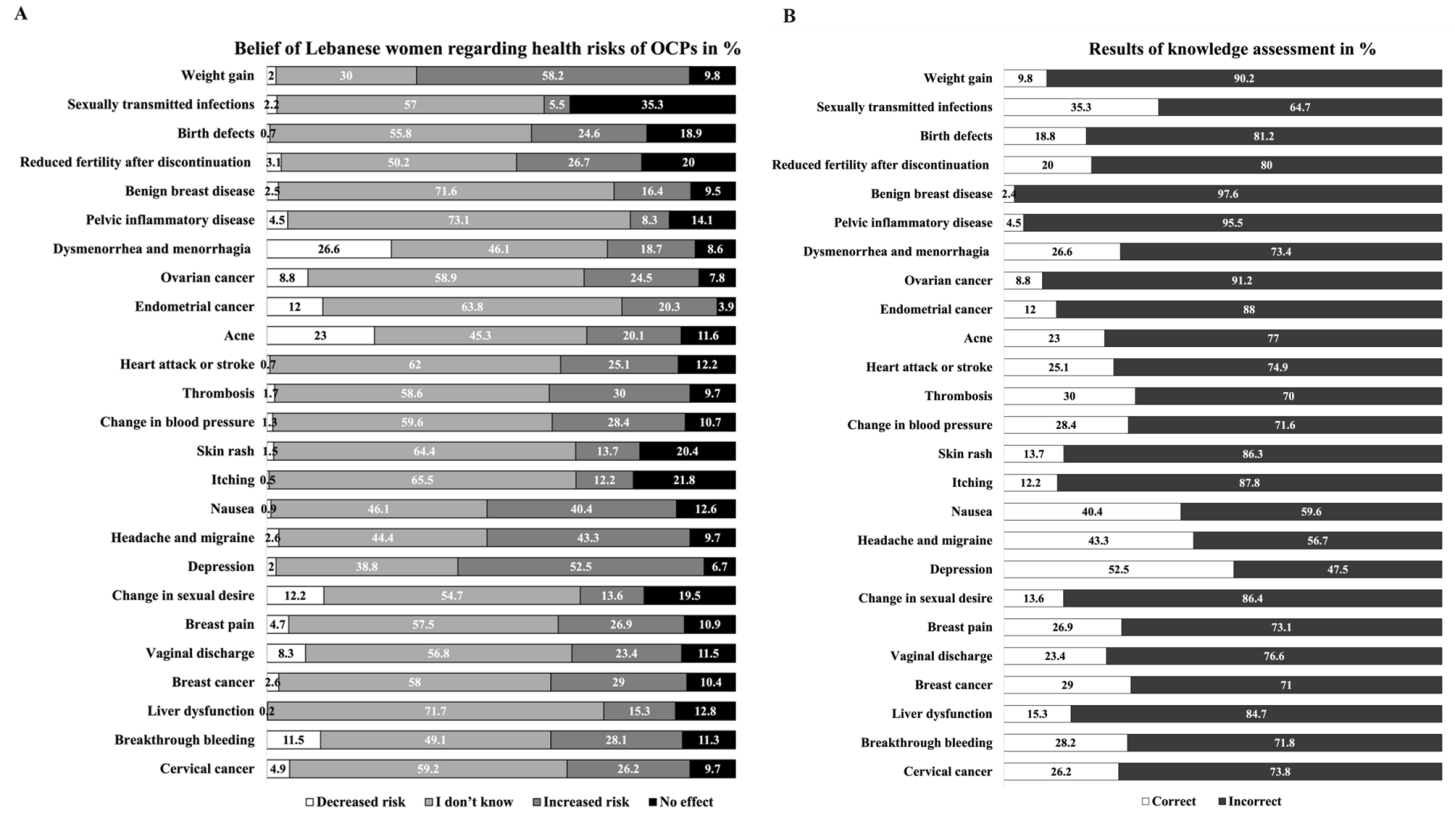
Self-assessment of knowledge of health benefits and health risks of OCPs (**A**) Beliefs of participants regarding the association between OCP use and 25 different medical conditions and (**B**) Percentage of participants who responded correctly to each item (N = 817)

Women were aware that OCP use increased the risk of depression (52%), nausea (40.4%), headaches and migraines (43.3%). However, when it comes to serious health risks, few women knew the increased risks of hypertension (28.4%), thromboembolism (25.1%), cervical cancer (26.2%), and breast cancer (29%) associated with OCP use. Similarly, few participants were aware of their non-contraceptive use against acne (23%), dysmenorrhea and menorrhagia (26.6%) as well as their protective role against endometrial cancer (12%), ovarian cancer (8.8%), pelvic inflammatory disease (4.5%), and benign breast disease (2.4%). The results of the self-assessment regarding the association between OCPs and different medical conditions are summarized in Fig. 2A and B.

### Correlates of knowledge

The results of the bivariate analyses are shown in Table 3. Higher OCP knowledge scores were seen in OCP users (mean = 7.05, SD = 4.7), young adults (mean = 6.64, SD = 5.14), single women (mean = 6.39, SD = 5.12), women without children (mean = 6.42, SD = 5.2), students (mean = 6.90, SD = 5.12), and participants with post-graduate degrees (mean = 6.13, SD = 4.85).

**Table 3:** Bivariate analysis of sociodemographic and clinical variables associated with OCP knowledge (N = 817)

*Variable*	*Mean knowledge score (SD)*	*p*	*Mean Risks Score /15 (SD)*	*p*	*Mean Benefits Score /6 (SD)*	*p*
**Ever used OCP**						
*Yes*	*7.05 (4.72)*	*< 0.001*	*4.83 (3.81)*	*< 0.001*	*1.10 (1.27)*	*< 0.001*
*No*	*4.73 (4.76)*		*3.55 (3.83)*		*0.54 (1.02)*	
**Age**						
*18–29*	*6.64 (5.14)*		*4.94 (4.02)*		*0.89 (1.22)*	
*30–49*	*4.91 (4.50)*	*< 0.001*	*3.33 (3.57)*	*< 0.001*	*0.69 (1.09)*	*0.024*
*50–64*	*4.64 (4.38)*		*3.25 (3.58)*		*0.62 (1.15)*	
**Marital status**						
*Single*	*6.39 (5.12)*	*< 0.001*	*4.73 (4.05)*	*< 0.001*	*0.89 (1.20)*	*0.009*
*Married*	*5.19 (4.67)*		*3.58 (3.68)*		*0.71 (1.15)*	
*Others*	*4.21 (3.50)*		*3.00 (3.02)*		*0.35 (0.73)*	
**Occupation**						
*Student*	*6.90 (5.12)*	*< 0.001*	*5.18 (4.01)*	*< 0.001*	*0.92 (1.26)*	*< 0.001*
*Unemployed*	*4.15 (3.97)*		*3.89 (3.90)*		*0.83 (1.19)*	
*Employed*	*5.66 (4.92)*		*2.99 (3.23)*		*0.47 (0.91)*	
**Governate**						
*Beirut*	*5.95 (4.97)*	*0.086*	*4.30 (3.97)*	*0.198*	*0.75 (1.16)*	*0.172*
*Bekaa and Baalbeck El Hermel*	*5.88 (5.33)*		*4.30 (3.83)*		*0.68 (1.20)*	
*Mount Lebanon*	*6.11 (4.93)*		*4.34 (4.03)*		*0.89 (1.18)*	
*North Lebanon and Akkar*	*5.51 (4.54)*		*3.97 (3.61)*		*0.68 (1.15)*	
*South Lebanon*	*4.66 (5.14)*		*3.36 (3.84)*		*0.67 (1.14)*	
**Educational level**						
*High School or less*	*4.87 (4.50)*	*0.007*	*3.71 (3.66)*	*0.205*	*0.50 (0.90)*	*< 0.001*
*Bachelor’s degree*	*5.97 (5.12)*		*4.28 (3.99)*		*0.84 (1.23)*	
Post-Grad Degree	*6.13 (4.85)*		*4.18 (3.92)*		*0.95 (1.26)*	
**Monthly income**						
*I prefer not to say*	*5.31 (4.85)*	*0.011*	*3.83 (3.80)*	*0.328*	*0.69 (1.11)*	*< 0.001*
*Less than 200$*	*5.15 (4.44)*		*3.92 (3.72)*		*0.57 (0.92)*	
*200$-400$*	*5.93 (5.47)*		*4.11 (4.00)*		*1.04 (1.48)*	
*400$-1000$*	*6.50 (4.68)*		*4.65 (3.93)*		*0.83 (1.20)*	
*> 1000$*	*6.85 (5.31)*		*4.40 (4.23)*		*1.15 (1.27)*	
**Parental status**						
*Has children*	*4.97 (4.40)*	*< 0.001*	*3.46 (3.55)*	*< 0.001*	*0.65 (1.11)*	*0.003*
*No children*	*6.42 (5.20)*		*4.70 (4.09)*		*0.90 (1.21)*	
**FH breast cancer**						
*Yes*	*5.87 (5.27)*	*0.298*	*4.21 (4.14)*	*0.555*	*0.80 (1.09)*	*0.944*
*No*	*5.73 (4.76)*		*4.08 (3.76)*		*0.77 (1.19)*	
*Not sure*	*4.82 (4.56)*		*3.62 (3.93)*		*0.76 (1.20)*	
**FH ovarian cancer**						
*Yes*	*5.64 (4.78)*	*0.996*	*4.18 (4.20)*	*0.978*	*0.60 (0.89)*	*0.127*
*No*	*5.70 (4.91)*		*4.07 (3.86)*		*0.76 (1.17)*	
*Not sure*	*5.68 (4.72)*		*4.10 (3.80)*		*1.00 (1.32)*	
**FH Endometrial cancer**						
*Yes*	*7.12 (5.12)*	*0.251*	*5.08 (3.88)*	*0.359*	*1.08 (1.32)*	*0.031*
*No*	*5.61 (4.87)*		*4.02 (3.87)*		*0.73 (1.14)*	
*Not sure*	*6.00 (4.83)*		*4.26 (3.89)*		*1.04 (1.29)*	
**FH Heart disease**						
*Yes*	*6.02 (5.04)*	*0.096*	*4.35 (3.72)*	*0.086*	*0.83 (1.22)*	*0.266*
*No*	*5.29 (4.64)*		*3.35 (4.00)*		*0.69 (1.09)*	
*Not sure*	*5.24 (4.70)*		*3.79 (3.62)*		*0.76 (1.14)*	

The results of the multivariable analysis are shown in Table 4.

**Table 4:** Factors associated independently with knowledge of OCPs (N = 817)

*Variable*	*Unstandardized B*	*Standardized B*	*95% CI*	*p-value*
**Model 1: Taking the knowledge score as the dependent variable** ^**1**^				
*Age (in years)*	*-0.891*	*-0.127*	*(-1.399, -0.383)*	*< 0.001*
OCP use (yes or no*)	*1.632*	*0.165*	*(1.046, 2.218)*	*< 0.001*
Student (yes or no*)	*2.240*	*0.212*	*(1.280, 3.199)*	*< 0.001*
Unemployed (yes or no*)	*-1.004*	*-0.102*	*(-1.728, -2.79)*	*0.007*
Governate (center or periphery*)	*0.575*	*0.059*	*(0.016, 1.134)*	*0.044*
FH of heart disease (yes or no*)	*-0.483*	*-0.065*	*(-0.901, --0.64)*	*0.024*
*Confidence in knowledge (five-point rating scale)*	*1.184*	*0.306*	*(0.933, 1.436)*	*< 0.001*
*Importance of receiving information (five-point rating scale)*	*0.382*	*0.077*	*(0.091, 0.673)*	*0.010*
*Satisfaction with information (five-point rating scale)*	*0.915*	*0.234*	*(0.667, 1.163)*	*< 0.001*
**Model 2: Taking the risk knowledge score as the dependent variable** ^**2**^				
*Age (in years)*	*-0.781*	*-0.141*	*(-1.198, -0.364)*	*< 0.001*
OCP use (yes or no*)	*0.822*	*0.104*	*(0.334, 1.309)*	*< 0.001*
Student (yes or no*)	*1.322*	*0.158*	*(0.689, 1.954)*	*< 0.001*
FH of heart disease (yes or no*)	*-0.437*	*-0.74*	*(-0.786, -0.088)*	*0.014*
*Confidence in knowledge (five-point rating scale)*	*0.851*	*0.277*	*(0.642, 1.061)*	*< 0.001*
*Importance of receiving information (five-point rating scale)*	*0.303*	*0.077*	*(0.063, 0.543)*	*0.014*
*Satisfaction with information (five-point rating scale)*	*0.649*	*0.209*	*(0.0442, 0.857)*	*< 0.001*
**Model 3: Taking the benefit knowledge score as the dependent variable** ^**3**^				
OCP use (yes or no*)	*0.467*	*0.197*	*(0.313, 0.621)*	*< 0.001*
Student (yes or no*)	*0.313*	*0.124*	*(0.096, 0.528)*	*0.005*
Education (bachelor degree vs. post-graduate degree vs. high school*)	*0.220*	*0.146*	*(0.121, 0.320)*	*< 0.001*
Parental status (yes or no*)	*-0.179*	*-0.077*	*(-0.372, 0.013)*	*0.068*
FH of endometrial cancer (yes or no*)	*0.095*	*0.075*	*(0.015, 0.176)*	*0.021*
*Confidence in knowledge (five-point rating scale)*	*0.161*	*0.174*	*(0.094, 0.227)*	*< 0.001*
*Satisfaction with information (five-point rating scale)*	*0.138*	*0.148*	*(0.072, 0.204)*	*< 0.001*

^1^Model 1: Dependent variable: total knowledge score of OCPs; Independent variables: age, OCP use, occupation, governorate of residence, family history of heart disease, confidence in knowledge, importance of receiving information and satisfaction with information

^2^Model 2: Dependent variable: risk knowledge score of OCPs; Independent variables: age, OCP use, occupation, family history of heart disease, confidence in knowledge, importance of receiving information and satisfaction with information

^3^Model 3: Dependent variable: benefit knowledge score of OCPs; Independent variables: OCP use, occupation, education, parental status, family history of endometrial cancer, confidence in knowledge, and satisfaction with information

^*^Reference

In the first model, the dependent variable is the total knowledge score of OCPs whereas the independent variables are the following sociodemographic factors: age, OCP use, occupation, governate of residence, family history of heart disease, confidence in knowledge, importance of receiving information and satisfaction with information.

Based on this model, living in central governates such as Beirut and Mount Lebanon (B = 0.575, p = 0.044), using OCPs (B = 1.632, *p* < 0.001), and being a student (B = 2.240, *p* < 0.001) were associated with a higher OCP knowledge score whereas older age (B = -0.891, *p* < 0.001), being unemployed (B = -1.004, *p* = 0.007), and having a family history of heart disease (B = -0.483, *p* = 0.024) were associated with a lower OCP knowledge score.

In addition, the knowledge score also increased for every unit increase in the confidence in OCP knowledge (B = 1.184, *p* < 0.001), the perceived importance of receiving information related to OCPs (B = 0.382, p = 0.01), and the satisfaction with the information related to OCPs (B = 0.915, *p* < 0.001).

In the second model, the dependent variable is the risk knowledge score of OCPs whereas the independent variables are the following sociodemographic factors: age, OCP use, occupation, family history of heart disease, confidence in knowledge, importance of receiving information and satisfaction with information.

Based on that model, OCP use (B = 0.822, *p* < 0.001) and being a student (B = 1.322, *p* < 0.001) were associated with a higher risk knowledge score, whereas older age (B= -0.781, *p* < 0.001) and having a family history of heart disease (B = -0.437, *p* = 0.014) were associated with a lower risk knowledge score. Results of the self-assessment (Fig. 2B) showed significantly high percentages of incorrect answers although most participants felt reasonably confident about their knowledge in OCPs.

The risk knowledge score also increased with the confidence in OCP knowledge (B = 0.851, *p* < 0.001), the perceived importance of receiving information related to OCPs (B = 0.303, *p* = 0.01), and the satisfaction with the information related to OCPs (B = 0.649, *p* < 0.001).

In the third model, the dependent variable is the benefit knowledge score of OCPs whereas the independent variables are the following sociodemographic factors: OCP use, level of education, parental status, family history of endometrial, confidence in knowledge and satisfaction with information.

For the that model, the results showed that OCP use (B = 0.467, *p* < 0.001), being a student (B = 0.313, *p* = 0.005), having a higher level of education (B = 0.220, *p* < 0.001), and having a family history of endometrial cancer (B = 0.095, *p* = 0.021) were associated with a higher benefit knowledge score (*p* < 0.001) whereas having children (B = -0.179, *p* = 0.068) was associated with a lower benefit knowledge score.

Similarly, the benefit knowledge score also increased with the confidence in OCP knowledge (B = 0.161, *p* < 0.001) and the same can be said about satisfaction with the information related to OCPs (B = 138, *p* < 0.001).

Collinearity was checked by examining the Correlation Matrix of the independent variables and high correlations [28] (close to 1 or -1) between pairs of variables were not detected. Moreover, based on the collinearity statistics report, the calculated VIF for each predictor in the model was around 1.0. Commonly, a VIF greater than 10 is often considered an indicator of problematic collinearity and it was not found in our results.

## Discussion

This study highlighted shortcomings in the knowledge of health benefits and risks related to OCPs among Lebanese women, despite OCPs being the most widely used contraceptive method. In addition, it identified several sociodemographic factors associated with better total, risk and benefit knowledge.

Our findings showed that most participants were aware of the association between OCP use and depression, nausea, headaches, and migraines which can be due to them being more commonly experienced and more spoken about among Lebanese women, as demonstrated by Abi Tayeh et al. [5] However, few women were able to identify important risks associated with OCPs like cardiovascular conditions and cancer. Similarly, few women were aware of the non-contraceptive benefits, even though a significant portion used them for such purposes. These findings are comparable to other studies conducted by Gaudet et al., Machado et al., and in Saudi Arabia [13,29]. It’s also important to consider that some medical conditions might not be well-understood by participants [14].

Similar to other studies, [14,30] a lot of participants believed the misconceptions associated with OCP use like infertility and weight gain, despite the lack of scientific evidence [22,23].

Furthermore, our study found that following the self-assessment, most participants indicated that they were unsatisfied with their knowledge but demonstrated a positive attitude given that the vast majority deemed receiving information related to OCPs extremely important.

These findings highlight the need to promote health education on overlooked health benefits and health risks but also to address common misconceptions.

Several variables were found to be independently associated with knowledge of the health benefits and risks of OCPs. For instance, women living in central Lebanese areas like Beirut and Mount Lebanon were found to be more knowledgeable about OCPs which can be due to easier access to sources of information and greater ability to afford OCPs [31]. Also, OCP users demonstrated greater knowledge scores which can be attributed to their personal experience or from information provided by physicians [6,19]. Furthermore, higher OCP benefit knowledge scores were found to be associated with higher educational levels and a family history of endometrial cancer. This can be explained by the fact that women with a higher education are more capable of grasping and applying gained information to promote good health [6,12,32,33]. Furthermore, participants with a family history of endometrial cancer are more likely to research their relatives’ illness and thus be aware of the non-contraceptive benefits of OCPs. Finally, confidence in OCP knowledge, the importance of receiving information about OCPs, and the satisfaction with information related to OCPs were correlated with higher knowledge scores, as seen with Philipson et al. [6]

In contrast, our study showed that variables like older age and unemployment were associated with lower OCP knowledge. This suggests that women past the reproductive age are less likely to be taking OCPs and therefore less informed about them [34]. However, this finding is contradicting with a study conducted by Al-Mass et al. in 2018 [24]. In addition, unemployed women with limited access to OCPs or lower income are less likely to be informed about OCPs [34]. Unexpectedly, women with a family history of heart disease appeared to be less knowledgeable about OCPs and this can be partially explained by the high prevalence of heart diseases in Lebanon which can lead to the underestimation of the seriousness of these diseases and the lack of awareness of the negative association with less standard risk factors such as OCP use. Parental status was also found to be associated with a lower benefit knowledge score; this suggests that women with children are no longer interested in family planning and thus unaware of the non-contraceptive benefits of OCPs [35].

Monthly income also showed a statistically significant correlation with the knowledge score (*p* = 0.011) and benefit knowledge score (p < 0.001). However, it was not included in the multivariable linear regression due to the ongoing economic collapse in Lebanon.

Finally, most participants did not discuss OCP use with healthcare professionals, despite considering them their main source of information. As such, this unilateral approach of doctor-patient relationship reveals a gap in OCP knowledge. To optimize a patient’s health decisions and treatment plan, there must be a two-sided relationship between the patient and their healthcare professional with both parties engaging in gaining knowledge and making informed decisions. Although it is the health professional’s responsibility to provide the necessary information, the patient must be willing to obtain and process what is provided to make sound decisions and be aware of the health benefits and risks of OCPs; this is known as health literacy [36,37]. Prior studies have demonstrated the inadequate health literacy among Lebanese women as well as its negative association with low education [38] and socioeconomic status [39]. Therefore, proper health literacy serves as a valuable complement to health professionals in providing the necessary information and securing a greater knowledge of the health benefits and risks of OCPs. Patients can increase their health literacy through several practical measures such as proactively seeking answers through medical consultations or other reliable sources. In addition, participating in campaigns can promote self-awareness, foster knowledge about OCPs and encourage discussions with other attendees and health professionals.

### Strength and limitations

To our knowledge, this study is the first to assess the knowledge of health benefits and risks of OCPs and their association with sociodemographic factors to address information needs among Lebanese women. A strong aspect of this study was its large sample size and the inclusion of women from different governates. Another important consideration is the well-constructed questionnaire that was used in a previous study conducted by Philipson et al. [6] which included important knowledge parameters. Furthermore, when analyzing key determinants that predict knowledge scores, 3 different models were computed for the total knowledge, health risks knowledge and health benefits knowledge scores, respectively.

The limitations of the study are inherent due to the cross-sectional design of our study. Although we had a relatively big sample size including women across Lebanon, yet the sample may not be representative of all the Lebanese population due to selection bias.

In addition, the distribution of the online questionnaire through social media may be limited to those with internet access.

## Conclusion

In conclusion, there’s poor knowledge about the health benefits and risks of OCPs especially among women with sociodemographic factors like older age, unemployment, a family history of heart disease, and parenthood. This limited awareness can influence both attitude and views, adversely affecting reproductive health.

In addition, health literacy plays a fundamental role in helping individuals understand health information, make informed decisions about their treatment plans, and communicate effectively with their healthcare providers.

As such, additional research should focus on assessing the impact of low OCP knowledge and low health literacy on reproductive health. This emphasizes the need to address this issue on a national level by implementing strategies to raise awareness and educate Lebanese women, while considering sociodemographic factors that might influence their beliefs.

Future prospective studies are warranted to evaluate the efficacy and outcomes of such strategies in improving the gap in knowledge of OCPs.

## Data Availability

The datasets used and/or analysed during the current study are available from the corresponding author on reasonable request.

## Abbreviations

OCP: Oral Contraceptive Pill
